# IMGT® Biocuration and Comparative Study of the T Cell Receptor Beta Locus of Veterinary Species Based on *Homo sapiens* TRB

**DOI:** 10.3389/fimmu.2020.00821

**Published:** 2020-05-05

**Authors:** Perrine Pégorier, Morgane Bertignac, Imène Chentli, Viviane Nguefack Ngoune, Géraldine Folch, Joumana Jabado-Michaloud, Saida Hadi-Saljoqi, Véronique Giudicelli, Patrice Duroux, Marie-Paule Lefranc, Sofia Kossida

**Affiliations:** IMGT®, The International ImMunoGeneTics Information System®, Centre National de la Recherche Scientifique (CNRS), Institut de Génétique Humaine (IGH), Université de Montpellier (UM), Montpellier, France

**Keywords:** IMGT, immunoinformatics, immunogenetics, T cell receptor, TRB locus

## Abstract

IMGT®, the international ImMunoGeneTics information system® is the global reference in immunogenetics and immunoinformatics. By its creation in 1989 by Marie-Paule Lefranc (Université de Montpellier and CNRS), IMGT® marked the advent of immunoinformatics, which emerged at the interface between immunogenetics and bioinformatics. IMGT® is specialized in the immunoglobulins (IG) or antibodies, T cell receptors (TR), major histocompatibility (MH), and proteins of the IgSF and MhSF superfamilies. T cell receptors are divided into two groups, αβ and γδ TR, which express distinct TR containing either α and β, or γ and δ chains, respectively. The TRβ locus (TRB) was recently described and annotated by IMGT® biocurators for several veterinary species, i.e., cat (*Felis catus*), dog (*Canis lupus familiaris*), ferret (*Mustela putorius furo*), pig (*Sus scrofa*), rabbit (*Oryctolagus cuniculus*), rhesus monkey (*Macaca mulatta*), and sheep (*Ovis aries*). The aim of the present study is to compare the genes of the TRB locus among these different veterinary species based on *Homo sapiens*. The results reveal that there are similarities but also differences including the number of genes by subgroup which may demonstrate duplications and/or deletions during evolution.

## 1. Introduction

IMGT®, the international ImMunoGeneTics information system®, http://www.imgt.org (1), is the global reference in immunogenetics and immunoinformatics (2), founded in 1989 by Marie-Paule Lefranc at Montpellier (Université de Montpellier and CNRS). IMGT® is a high-quality integrated knowledge resource specialized in the immunoglobulins (IG) or antibodies, T cell receptors (TR), major histocompatibility (MH) of human and other vertebrate species, and in the immunoglobulin superfamily (IgSF), MH superfamily (MhSF) and related proteins of the immune system (RPI) of vertebrates and invertebrates.

T cell receptors are divided into two groups, αβ and γδ TR, which express distinct TR containing either α and β, or γ and δ chains, respectively. TR comprise a variable and a constant domain. The variable domain is the result of one rearrangement between variable (V) and joining (J) genes for α and γ chains, and two consecutive rearrangements between diversity (D) and J genes then between V and partially rearranged D-J genes for β and δ chains. After transcription, the V–(D)–J sequence is spliced to the constant (C) gene to give the final transcript (3).

The human TRβ locus (TRB) consists of a cluster of TRBV genes located upstream (in 5′) of two D-J-C clusters, each composed of one TRBD, six to eight TRBJ and one TRBC, followed by a single TRBV in inverted transcriptional orientation which rearranges by a mechanism of inversion (3). A gene family, the protease serine (PRSS) trypsinogen genes (TRY), is situated among the TRBV genes. The IMGT 5′ borne of the TRB locus is the monooxygenase dopamine-beta-hydroxylase-like 2 (MOXD2) gene and the IMGT 3′ borne of the locus is the ephrin type-b receptor 6 (EPHB6) gene. These two genes were defined as IMGT borne of the TRB locus because they correspond to genes (other than IG or TR) located, respectively, in the 5′ and 3′ end of the locus and they are conserved among species (http://imgt.org/IMGTindex/IMGTborne.php).

Animal species, mice as well as large animals, are essential model for the biological research and studies on farm animals for example, greatly contribute to fundamental and applied immunology (4). Furthermore, several veterinary species are useful for biotechnological applications that can also be applied to human medicine. This justifies the interest of scientists in the genomic organization of locus of genes involved in the immune response, notably the TRB locus for veterinary species. In this study, we compare the TRB locus of seven veterinary species namely cat (*Felis catus*), dog (*Canis lupus familiaris*), ferret (*Mustela putorius furo*), pig (*Sus scrofa*), rabbit (*Oryctolagus cuniculus*), rhesus monkey (*Macaca mulatta*), and sheep (*Ovis aries*) against the human (*Homo sapiens*) locus. The rhesus monkey, widely used as a model to study infection and immunity (5, 6) due to its genetic relationship with humans, is used for the development and testing of vaccines as is the rabbit (7), although evolutionarily closer to mouse than to human. The cat is for example a model for the study of the immunodeficiency virus due to the similarities between the feline immunodeficiency virus and the human one (8, 9), and the dog is a reliable model for the immune response during the development (10, 11). The ferret is an animal model of predilection for the pathogenesis of different respiratory viruses (12) as it has a lung physiology similar to that of human (13). Sheep is also a valuable model to study respiratory disorders as allergic asthma during pregnancy in relation with lung and immune development (14). Finally, T and B Cell immune responses to Influenza viruses were studied in pig (15), which represents also one of the large animal model for human cancer vaccine development (16).

The aim of this study is to present the methodology and results of a comparative study of the TRB locus among these seven veterinary species using human as reference.

## 2. Materials and Methods

### 2.1. Annotation of the TRB Locus

**Figure 1:**
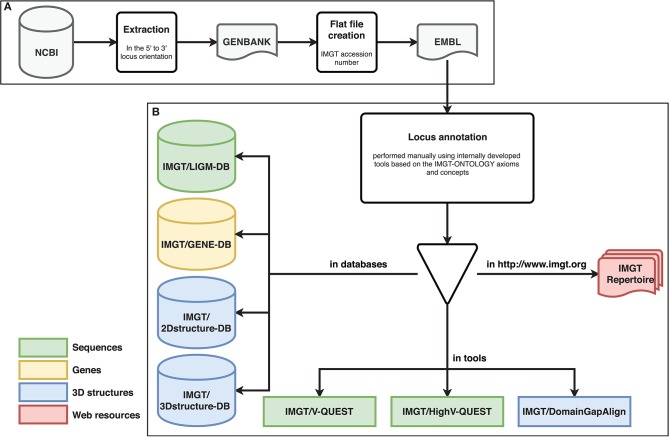
Different steps of biocuration pipeline. Databases are shown as cylinders, tools as rectangles and web resources as red documents. **(A)** Extraction and preparation of the locus sequences. **(B)** Locus annotation and data entry in the IMGT® reference directory used in IMGT® databases and tools.

Each locus sequence was localized on the corresponding chromosome, when available, or on the scaffolds and subsequently extracted from NCBI assembly (17) in GenBank format. The locus orientation on a chromosome can be either forward (FWD) or reverse (REV) therefore the REV locus sequences were placed in the 5′ to 3′ locus orientation. Each locus sequence was assigned to an IMGT® accession number (dog: IMGT000005, rhesus monkey: IMGT000012, ferret: IMGT000022 and IMGT000023, rabbit: IMGT000032, cat: IMGT000037, pig: IMGT000039, and sheep: IMGT000042). The ferret has two accession numbers because the locus sequences belong to two different unplaced scaffolds (*cf*. Figure 1A).

The biocuration has been performed manually assisted by internally developed tools [IMGT/LIGMotif (18), NtiToVald and IMGT/Automat (19)] based on the IMGT-ONTOLOGY axioms and concepts: “IDENTIFICATION,” “DESCRIPTION,” “CLASSIFICATION,” “NUMEROTATION,” “LOCALIZATION,” “ORIENTATION,” and “OBTENTION” (20). IMGT-ONTOLOGY includes the controlled vocabulary and annotation rules which are indispensable to ensure accuracy, consistency and coherence.

The nomenclature of all TRBV genes, “CLASSIFICATION” axiom of IMGT-ONTOLOGY, was characterized according to the human TRBV genes using Clustal Omega (21) and NGPhylogeny.fr (22) [using MAFFT (23) and PhyML (24) programs] to define the subgroups, except for the TRBV1 subgroup. TRBV genes are designated by a number for the subgroup followed, whenever there are several genes belonging to the same subgroup, by a hyphen and a number picturing their relative localization in the locus. Numbers increase from 5′ to 3′ in the locus (3). Two genes belong to the same subgroup if their identity percentage is >75% in their V-REGION.

The functionality of the genes was defined according to the IMGT “functionality” concept, part of the “IDENTIFICATION” axiom of IMGT-ONTOLOGY, described in http://imgt.org/IMGTScientificChart/Sequ enceDescription/IMGTfunctionality.html.

The main concept of the “DESCRIPTION” axiom of IMGT-ONTOLOGY correspond to IMGT® standardized labels in the databases and tools. A set of specific labels was defined to describe the different organizations of IG and TR genes in clusters at the scale of the locus or of the chromosome. They are available from the IMGT/LIGM-DB database, http://www.imgt.org/ligmdb/label#. More than 300 IMGT® standardized labels were precisely defined for sequences.

The standardized annotation allows data entry in the IMGT® reference directory used in IMGT® databases and tools [IMGT/LIGM-DB (25), IMGT/GENE-DB (26), IMGT/3Dstructure-DB and IMGT/2Dstructure-DB (27), IMGT/V-QUEST (28), IMGT/HighV-QUEST (29), and IMGT/DomainGapAlign (30)] (*cf*. Figure 1B). IMGT® genomic annotated data are then synthesized in IMGT Repertoire (http://imgt.org/IMGTrepertoire/) including several organized web pages (Locus representation, Locus description, Locus in genome assembly, Locus gene order, Gene tables, Potential germline repertoire, Protein displays, Alignments of alleles, Colliers de Perles (31, 32), and [CDR1-IMGT.CDR2-IMGT.CDR3-IMGT] lengths) (*cf*. Figure 1B).

### 2.2. Comparison of the TRB Locus

The expertised data obtained by biocuration were compared to human TRB locus. The human TRB locus is located on chromosome 7 (7q34) on FWD orientation and spans 620 kilobases (kb). The IMGT 5′ borne (MOXD2) has been identified 52 kb upstream of the first gene of the locus and the IMGT 3′ borne (EPHB6), 41 kb downstream (in 3′) of the last gene of the locus. The potential repertoire consists of 65-68 TRBV genes due to polymorphism by insertion/deletion [41–43 functional (F), 6 ORF, 13–14 pseudogenes (P), 1 F or ORF and 4 F or P (depending on alleles)] belonging to 33 TRBV subgroups, 2 TRBD genes (F), 14 TRBJ genes (12 F, 1 ORF and 1 F or ORF), and 2 TRBC genes (F) (3, 33, 34).

A comparison was performed based on the number of genes in the locus as well as the number of genes per subgroup (potential germline repertoire), the locus representation, the functionality of genes and the CDR lengths. Potential duplications and/or deletions that may have occurred during evolution are susceptible to be highlighted from this sort of comparisons.

## 3. Results

### 3.1. Annotation of TRB Loci

The seven TRB loci were annotated following the previously described pipeline (*cf*. Figure 1). The results of the annotation described below are summarized in Table 1. The information regarding the genome assemblies and the IMGT bornes is provided in Table S1.

**Table 1:** Results of the analysis of TRB loci in human (*Homo sapiens*), rhesus monkey (*Macaca mulatta*), dog (*Canis lupus familiaris*), cat (*Felis catus*), ferret (*Mustela putorius furo*), rabbit (*Oryctolagus cuniculus*), sheep (*Ovis aries*), and pig (*Sus scrofa*).

**Species**	**Chromosome orientation**	**Size (kb)**	**Number of TRBV genes**	**Number of TRBD genes**	**Number of TRBJ genes**	**Number of TRBC genes**
*Homo sapiens*	7 (FWD)	620	65-68	2	14	2
*Macaca mulatta*	3 (FWD)	736	77	2	14	2
*Canis lupus familiaris*	16 (REV)	271	36	2	12	2
*Felis catus*	A2 (FWD)	302	33	2	12	2
*Mustela putorius furo*	Unplaced	260	34	2	12	2
*Oryctolagus cuniculus*	Unplaced	543	77	2	12	2
*Ovis aries*	4 (FWD)	506	94	3	19	3
*Sus scrofa*	18 (REV)	407	38	3	20	3

*Data available in IMGT Repertoire (IG and TR) http://imgt.org/IMGTrepertoire/ > Locus and genes > Locus descriptions > Locus description > TRB > Human, ibid. Rhesus monkey, ibid. Dog, ibid. Cat, ibid. Ferret, ibid. Rabbit, ibid. Sheep, ibid. Pig*.

The rhesus monkey TRB locus, on chromosome 3 (FWD), spans 736 kb and consists of 77 TRBV genes (51 F, 6 ORF, 16 P, 3 F or P and 1 ORF or P) belonging to 32 TRBV subgroups, 2 TRBD genes (F), 14 TRBJ genes (13 F and 1 P), and 2 TRBC genes (1 F and 1 F or P) (35). 7 new genes (5 TRBV and 2 TRBC) have been annotated compared to the article. The IMGT 5′ borne (MOXD2) has been identified 75 kb upstream of the first gene of the locus and the IMGT 3′ borne (EPHB6), 48 kb downstream of the last gene of the locus.

The dog TRB locus, on chromosome 16 (REV), spans 271 kb and consists of 36 TRBV genes (22 F, 1 ORF and 13 P) belonging to 25 TRBV subgroups, 2 TRBD genes (F), 12 TRBJ genes (9 F, 2 ORF and 1 P), and 2 TRBC genes (F) (36, 37). 1 described gene (TRBV2-4) has not been annotated because it doesn't have criteria to be considered as TRBV gene and 3 genes (TRBV26, TRBV28, and TRBJ2-1) have changed their functionality compared to the article. The IMGT 5′ borne (MOXD2) has been identified 4 kb upstream of the first gene of the locus and the IMGT 3′ borne (EPHB6), 36 kb downstream of the last gene of the locus.

The cat TRB locus, on chromosome A2 (FWD), spans 302 kb and consists of 33 TRBV genes (20 F, 4 ORF and 9 P) belonging to 27 TRBV subgroups, 2 TRBD genes (F), 12 TRBJ genes (8 F, 1 ORF and 3 P), and 2 TRBC genes (F) (38). The IMGT 5′ borne (MOXD2) has been identified 4 kb upstream of the first gene of the locus and the IMGT 3′ borne (EPHB6), 30 kb downstream of the last gene of the locus.

The ferret TRB locus, unplaced, spans 260 kb and consists of 34 TRBV genes (20 F, 3 ORF and 11 P) belonging to 28 TRBV subgroups, 2 TRBD genes (F), 12 TRBJ genes (7 F, 4 ORF and 1 P), and 2 TRBC genes (F) (39). 7 new genes (TRBV2, TRBVA, TRBV5-1, TRBV5-3, TRBV11, TRBV17, and TRBV23) have been annotated and 4 genes (TRBV1, TRBV6, TRBV20, and TRBJ1-1) have changed their functionality compared to the article. The IMGT 5′ borne (MOXD2) has been identified 5 kb upstream of the first gene of the locus and the IMGT 3′ borne (EPHB6), 39 kb downstream of the last gene of the locus.

The rabbit TRB locus, unplaced, spans 543 kb and consists of 77 TRBV genes (59 F, 17 P and 1 F or P) belonging to 26 TRBV subgroups, 2 TRBD genes (F), 12 TRBJ genes (11 F and 1 ORF), and 2 TRBC genes (F) (40). 2 new genes (TRBV9-1 and TRBV17) have been annotated and 3 genes have changed their nomenclature (TRBV5-17 became TRBV9-2, as a consequence, TRBV5-18 became TRBV5-17 and TRBV9 became TRBV13) compared to the article. The IMGT 5′ borne (MOXD2) has been identified 3 kb upstream of the first gene of the locus and the IMGT 3′ borne (EPHB6), 51 kb downstream of the last gene of the locus.

The sheep TRB locus, on chromosome 4 (FWD), spans 506 kb and consists of 94 TRBV genes (46 F, 12 ORF and 36 P) belonging to 26 TRBV subgroups, 3 TRBD genes (F), 19 TRBJ genes (17 F, 1 ORF and 1 P), and 3 TRBC genes (F) (41, 42). 95 new gene (addition of TRBV genes and TRBJ3-6) have been annotated compared to the article. The IMGT 5′ borne (MOXD2) has been identified 6 kb upstream of the first gene of the locus and the IMGT 3′ borne (EPHB6), 53 kb downstream of the last gene of the locus.

The pig TRB locus, on chromosome 18 (REV), spans 407 kb and consists of 38 TRBV genes (27 F and 11 P) belonging to 24 TRBV subgroups, 3 TRBD genes (F), 20 TRBJ genes (18 F and 2 P), and 3 TRBC genes (F) (43). 2 genes (TRBV28 and TRBJ3-6) have changed their functionality compared to the article. The IMGT 5′ borne (MOXD2) has been identified 3.5 kb upstream of the first gene of the locus and the IMGT 3′ borne (EPHB6), 49 kb downstream of the last gene of the locus.

The differences observed between the data indicated in the articles and the data expertised by IMGT® (*cf*. Table S2) correspond to the fact that the articles are, in general, published before the expertise of IMGT® biocurators. The additional genes found during the fine annotation (either TRBV or TRBJ) correspond to very mutated pseudogenes (insertions/deletions in the coding region, absence of motifs, etc.) and the functionalities are revised according to the rules defined by biocurators (*cf*. http://imgt.org/IMGTScientificChart/SequenceDescription/IMGTfunctionality.html#P1-2).

### 3.2. Comparison of the TRBV Genes

**Figure 2:**
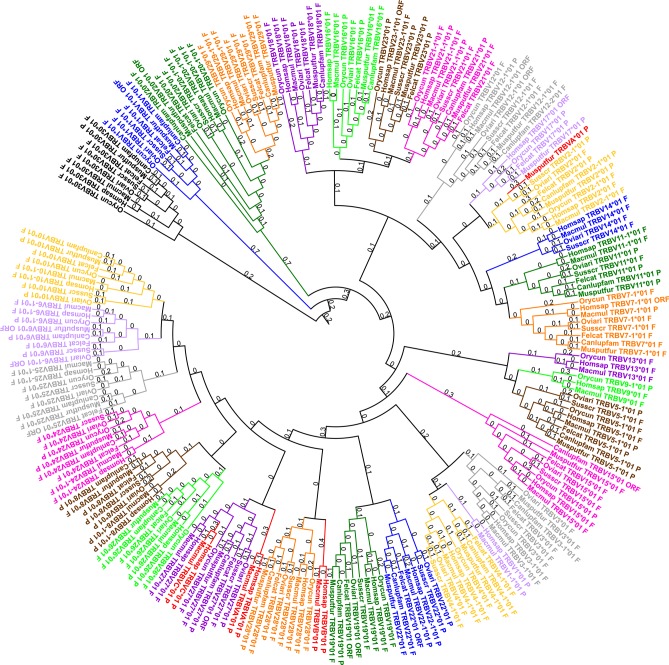
Phylogenetic tree of all TRBV subgroups for all species with one representative gene per subgroup (using V-REGION). Homsap: human, Macmul: rhesus monkey, Canlupfam: dog, Felcat: cat, Musputfur: ferret, Orycun: rabbit, Oviari: sheep, and Susscr: pig. The different colors highlight the different subgroups. In red: highly degenerated pseudogenes (TRBVA, TRBVB and TRBVC) included in others subgroups. Tree generated using NGPhylogeny.fr (22) (with MAFFT (23) and PhyML (24) programs) and iTOL v4 (44).

All subgroups were defined according to those of the human genome, with the exception of the TRBV1 subgroup. A phylogenetic tree with one representative gene by subgroup for the seven species studied was created in order to highlight the distance between the different species within a subgroup (*cf*. Figure 2). This phylogenetic tree shows that, for the seven species, the genes of a subgroup are grouped in the same branch with a corresponding human gene. Only TRBVA, TRBVB, and TRBVC, highly degenerated pseudogenes present only in human, rhesus monkey and ferret for the TRBVA, are included in other subgroups. Some subgroups are very close, in particular the subgroups TRBV9 and TRBV5 which are intermingled (*cf*. Figure S1). However, there is <75% identity between the genes of these two subgroups for a given species, so they cannot be considered as genes belonging to the same subgroup.

The number of TRBV genes varies depending on the species. On average, there are between 33 and 38 TRBV in dog, cat, ferret and pig. There are between 65 and 68 TRBV in humans (depending on insertion/deletion polymorphism), 77 TRBV in rhesus monkey and rabbit and 94 TRBV in sheep (*cf*. Table 1). The number of genes per subgroup also varies according to the species (*cf*. Table 2). TRBV5, TRBV6, and TRBV7 subgroups are the most represented in humans and rhesus monkey (~10 genes per subgroup). These are also the most represented subgroups in rabbit (with 17 TRBV5, 14 TRBV6, and 14 TRBV7). In sheep, only the TRBV5 and TRBV6 subgroups are highly represented (about 30 genes for each subgroup). TRBV1 to TRBV12 subgroups are those which contain several genes per subgroup with a number varying according to the species. In contrast, there is only one gene per subgroup for subgroups from TRBV13 to TRBV30 except for the TRBV20 subgroup in rabbit and pig (2 and 3 genes, respectively) and the TRBV21 subgroup in rabbit and sheep (7 and 6 genes, respectively). In addition, some subgroups are absent in several species, such as subgroups TRBV9, TRBV13 and TRBV14 in dog, cat and ferret, and subgroups TRBV9 and TRBV13 in sheep and pig for example.

**Table 2:** IMGT Potential germline repertoires of the TRBV subgroups in human (*Homo sapiens*), rhesus monkey (*Macaca mulatta*), dog (*Canis lupus familiaris*), cat (*Felis catus*), ferret (*Mustela putorius furo*), rabbit (*Oryctolagus cuniculus*), sheep (*Ovis aries*), and pig (*Sus scrofa*).

**Subgroups**	***Homo sapiens***	***Macaca mulatta***	***Canis lupus familiaris***	***Felis catus***	***Mustela putorius furo***	***Oryctolagus cuniculus***	***Ovis aries***	***Sus scrofa***
TRBV1	1 P	3 P	1 F	1 F	1 O	1 F	1 F	1 F
TRBV2	1 F (3)	3 F	3 P	1 P	1 P	1 F, 1 FP (2)	1 F	1 F, 4 P
TRBV3	1 F (2), 1 P (3)	4 F	2 F, 1 P	1 F	1 F	1 F (2)	1 F	1 F
TRBV4	3 F (8)	3 F	4 F	2 F	2 F	1 F (2)	1 F	4 F, 1 P
TRBV5	5 F (12), 2 O (3), 1 P	5 F (6), 1 O (2), 2 P (4), 2 FP (4)	2 F, 2 P	3 F, 1 P	2 F, 2 P	13 F, 4 P	14 F, 5 O, 14 P	2 F, 1 P
TRBV6	8 F (13), 1 O	5 F (8), 3 O, 1 P (2), 1 OP (2)	1 P	1 F	1 O	13 F, 1 P	10 F, 5 O, 15 P	1 P
TRBV7	5 F (18), 1 O, 1 P (2), 1 FO (5), 1 FP (2)	6 F (7), 1 O, 3 P, 1 FP (2)	1 F	2 F	2 F	9 F, 5 P	2 F	2 F
TRBV8	2 P (4)	2 P (3)	1 P	1 P	1 F	-	1 P	1 P
TRBV9	1 F (3)	1 F (2)	-	-	-	2 P	-	-
TRBV10	2 F (6), 1 FP (3)	3 F (5)	1 F	1 F	1 P	1 F	1 P	1 F
TRBV11	3 F (8)	3 F	1 P	1 P	1 P	-	1 P	1 F
TRBV12	3 F (4), 2 P	3 F, 1 O (4)	1 F, 1 P	1 F, 1 P	2 F	1 F	1 F, 1 O	1 F, 1 P
TRBV13	1 F (2)	1 F	-	-	-	1 F	-	-
TRBV14	1 F (2)	1 F	-	-	-	-	1 F	1 F
TRBV15	1 F (3)	1 F	1 O	1 F	1 F	1 P	1 F	1 F
TRBV16	1 FP (3)	1 F	1 F	1 P	1 F	1 P	1 F	-
TRBV17	1 O (2)	-	-	1 P	1 P	1 P	-	-
TRBV18	1 F	1 F	1 F	1 F	1 F	1 F	1 P	-
TRBV19	1 F (3)	1 F	1 P	1 O	1 F	1 F	1 O	1 F
TRBV20	1 F (7)	1 F	1 F	1 F	1 O	2 F	1 F	3 F
TRBV21	1 P (2)	1 F	1 P	1 F	1 F	7 F	5 F, 1 P	1 F
TRBV22	1 P	1 P	1 F	1 O	1 F	-	1 P	1 P
TRBV23	1 O	1 F	-	1 P	1 P	1 P	-	1 P
TRBV24	1 F (2)	1 F	1 F	1 F	1 P	1 P	1 F	1 F
TRBV25	1 F	1 F	1 F	1 O	1 F	1 F	1 F	1 F
TRBV26	1 P	1 P	1 F	1 F	1 F	1 F	1 F	-
TRBV27	1 F	1 F	1 P	1 O	1 F	1 F	1 P	1 F
TRBV28	1 F	1 F	1 F	1 P	1 P	1 F	1 F	1 F
TRBV29	1 F (3)	1 F	1 F	1 F	1 F	1 F	1 F	1 F
TRBV30	1 FP (5)	1 F	1 F	1 F	1 P	1 F	1 F	1 F
TRBVA	1 P (2)	1 P	-	-	1 P	-	-	-
TRBVB	1 P (2)	1 P	-	-	-	-	-	-
TRBVC	1 P	1 P	-	-	-	-	-	-
Total per Fct	43 F + 6 O + 14 P + 4 FP + 1 FO	51 F + 6 O + 16 P + 3 FP + 1 OP	22 F + 1 O + 13 P	20 F + 4 O + 9 P	20 F + 3 O + 11 P	59 F + 17 P + 1 FP	46 F + 12 O + 36 P	27 F + 11 P
Total genes	68 (151)	77 (96)	36	33	34	77 (80)	94	38

*For each TRBV subgroup, in each species, the number of TRBV genes by functionality and, between parentheses, the number of alleles are shown. F: functional; O: ORF; P: pseudogene; FO, FP, OP: genes with alleles of different functionalities. Data available in IMGT Repertoire (IG and TR) http://imgt.org/IMGTrepertoire/ > Locus and genes > Potential germline repertoires > TRBV, TRBD, TRBJ > Human, ibid. Rhesus monkey, ibid. Dog, ibid. Cat, ibid. Ferret, ibid. Rabbit, ibid. Sheep, ibid. Pig*.

**Figure 3:**
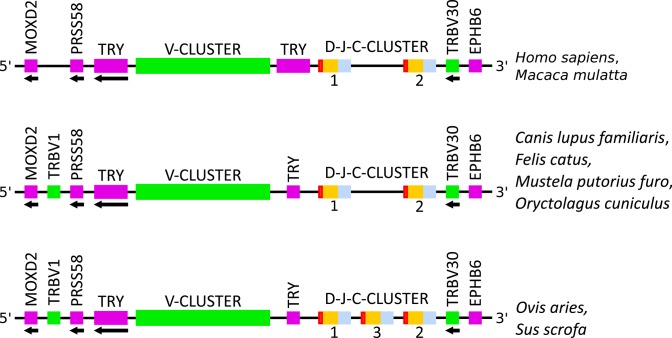
Schematic comparison of the TRB locus, not to scale, among human (*Homo sapiens*), rhesus monkey (*Macaca mulatta*), dog (*Canis lupus familiaris*), cat (*Felis catus*), ferret (*Mustela putorius furo*), rabbit (*Oryctolagus cuniculus*), sheep (*Ovis aries*), and pig (*Sus scrofa*). Colors are according to IMGT color menu for regions and domains (http://imgt.org/IMGTScientificChart/RepresentationRules/colormenu.php#h1_31): in pink: genes not related, in green: TRBV genes, in red: TRBD genes, in orange: TRBJ genes and in blue: TRBC genes. TRY: trypsinogenes. Data available in IMGT Repertoire (IG and TR) http://imgt.org/IMGTrepertoire/ > Locus and genes > Locus representations > TRB > Human, *ibid*. Rhesus monkey, *ibid*. Dog, *ibid*. Cat, *ibid*. Ferret, *ibid*. Rabbit, *ibid*. Sheep, *ibid*. Pig.

**Figure 4:**
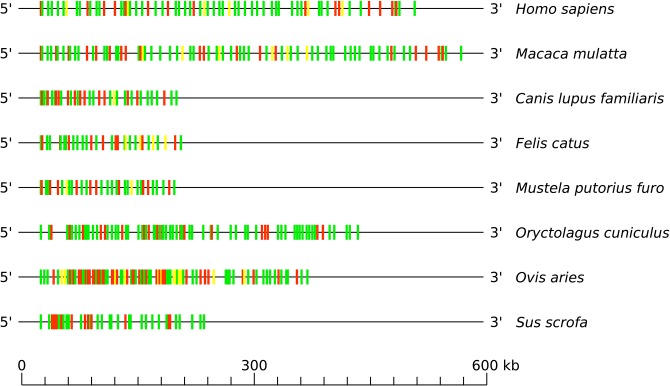
Schematic comparison of the TRB V-CLUSTER among human (*Homo sapiens*), rhesus monkey (*Macaca mulatta*), dog (*Canis lupus familiaris*), cat (*Felis catus*), ferret (*Mustela putorius furo*), rabbit (*Oryctolagus cuniculus*), sheep (*Ovis aries*), and pig (*Sus scrofa*). Colors are according to IMGT color menu for genes (http://imgt.org/IMGTScientificChart/RepresentationRules/colormenu.php#h1_28): in green: functional genes, in yellow: ORF genes and in red: pseudogenes. Data available in IMGT Repertoire (IG and TR) http://imgt.org/IMGTrepertoire/ > Locus and genes > Locus representations > TRB > Human, *ibid*. Rhesus monkey, *ibid*. Dog, *ibid*. Cat, *ibid*. Ferret, *ibid*. Rabbit, *ibid*. Sheep, *ibid*. Pig.

By consequence, the size of the V-CLUSTER (which describes the principal set of TRBV genes) (*cf*. Figure 3) varies (*cf*. Figure 4). The V-CLUSTER is more extensive in human (68 genes on 530 kb) and rhesus monkey (77 genes on 580 kb) than in the cat, dog, ferret, and pig, which is consistent with the number of genes in these species (around 35 genes over 200–250 kb). In contrast, the V-CLUSTER of the sheep, the species with the largest number of genes (94), is less extensive (lower than 400 kb) which indicates a higher gene density. Similarly for the rabbit which has the same number of genes as the rhesus monkey over a shorter length by 150 kb. Regarding the functionality of TRBV genes, the proportion of functional genes is well-conserved among human, rhesus monkey, cat, dog, ferret and pig. However, it is greater than in rabbit and much lower in sheep, the species in which there are more pseudogenes (*cf*. Figure 4 and Table 2).

**Figure 5:**
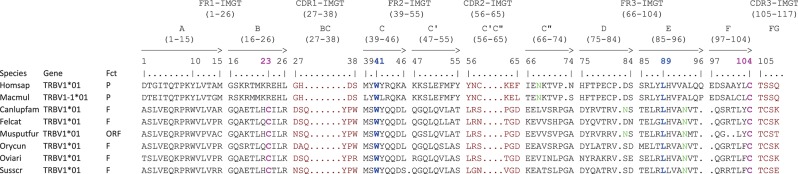
Protein display of the TRBV1 gene in human (Homsap), rhesus monkey (Macmul), dog (Canlupfam), cat (Felcat), ferret (Musputfur), rabbit (Orycun), sheep (Oviari), and pig (Susscr). The description of the strands and loops is according to the IMGT unique numbering for V-REGION (45). Data available in IMGT Repertoire (IG and TR) http://imgt.org/IMGTrepertoire/ > Proteins and alleles > Protein displays > V-REGION > TRBV > Human, *ibid*. Rhesus monkey, *ibid*. Dog, *ibid*. Cat, *ibid*. Ferret, *ibid*. Rabbit, *ibid*. Sheep, *ibid*. Pig.

Another difference among the species concerns the TRBV1 gene which is localized before PRSS58 in several species (*cf*. Figure 3). This gene is the only one for which the nomenclature in cat, dog, ferret, pig, rabbit and sheep does not correspond with that of human. In fact, the TRBV1 gene present in human has not been found in these species and inversely, the TRBV1 of these species is found neither in human nor in rhesus monkey. This is why the sequence of this gene is different according to its localization (*cf*. Figure 5). In the species where TRBV1 is localized upstream of PRSS58, the CDR1-IMGT is longer [2 additional amino acids (AA)] and there is a deletion of two AA between positions 96 and 97 in FR3-IMGT according to the IMGT unique numbering for V-REGION (45) (*cf*. Figure 5 and Table 3).

**Table 3:** CDR lengths by subgroup and species in human (*Homo sapiens*), rhesus monkey (*Macaca mulatta*), dog (*Canis lupus familiaris*), cat (*Felis catus*), ferret (*Mustela putorius furo*), rabbit (*Oryctolagus cuniculus*), sheep (*Ovis aries*), and pig (*Sus scrofa*).

**Subgroups**	***Homo sapiens***	***Macaca mulatta***	***Canis lupus familiaris***	***Felis catus***	***Mustela putorius furo***	***Oryctolagus cuniculus***	***Ovis aries***	***Sus scrofa***
TRBV1	[4.6.4]	[4.6.4]	[6.6.4]	[6.6.4]	[6.6.4]	[6.6.4]	[6.6.4]	[6.6.4]
TRBV2	[5.6.4]	[5.6.4]	-	-	-	[5.6.4]	[5.6.4]	[5.6.4]
TRBV3	[5.6.4]	[5.6.4]	[5.6.4]	[5.6.4]	[5.6.4]	[5.6.4]	[5.6.4]	[5.6.4]
TRBV4	[5.6.4]	[5.6.4]	[5.6.4]	[5.6.4]	[5.6.4]	[5.6.4]	[5.6.4]	[5.6.4]
TRBV5	[5.6.4]	[5.6.4]	[5.6.4]	[5.6.4]	[5.6.4]	[5.6.4]	[0.6.4] [5.6.4]	[5.6.4]
TRBV6	[5.6.4]	[5.6.4]	-	[5.6.4]	[5.10.4]	[5.6.3] [5.6.4]	[5.6.4]	[5.6.4]
TRBV7	[5.6.4]	[5.6.4]	[5.6.4]	[5.6.4]	[5.6.4]	[5.6.4]	[5.6.4]	[5.6.4]
TRBV8	-	-	-	-	[5.6.4]	-	-	-
TRBV9	[5.6.4]	[5.6.4]	-	-	-	-	-	-
TRBV10	[5.6.4]	[5.6.4]	[5.6.4]	[5.6.4]	-	[5.6.4]	-	[5.6.4]
TRBV11	[5.6.4]	[5.6.4]	-	-	-	-	[5.6.4]	[5.6.4]
TRBV12	[5.6.4]	[5.6.4]	[5.6.4]	[5.6.4]	[5.6.4]	[5.6.4]	[5.6.4]	[5.6.4]
TRBV13	[5.6.4]	[5.6.4]	-	-	-	[5.6.4]	-	-
TRBV14	[5.6.4]	[5.6.4]	-	-	-	-	[5.6.4]	[5.6.4]
TRBV15	[5.6.4]	[5.6.4]	[5.6.4]	[5.6.4]	[5.6.4]	-	[5.6.4]	[5.6.4]
TRBV16	[5.6.4]	[5.6.4]	[5.6.4]	-	[5.6.4]	-	[5.6.4]	-
TRBV17	[5.6.3]	-	-	-	-	-	-	-
TRBV18	[5.6.4]	[5.6.4]	[5.6.4]	[5.6.4]	[5.6.4]	[5.6.4]	-	-
TRBV19	[5.6.4]	[5.6.4]	[5.6.4]	[5.6.4]	[5.6.4]	[5.6.4]	[5.6.4]	[5.6.4]
TRBV20	[6.7.3]	[6.7.3]	[6.7.3]	[6.7.3]	[6.7.2]	[6.7.3]	[6.7.3]	[6.7.3]
TRBV21	[5.6.4]	[5.6.3]	[5.6.4]	[5.6.4]	[5.6.4]	[5.6.4]	[5.6.4]	[5.6.4]
TRBV22	-	[3.6.4]	[5.6.4]	[5.6.4]	[5.6.4]	-	[5.6.3]	-
TRBV23	[5.6.4]	[5.6.4]	-	-	-	-	-	-
TRBV24	[5.6.5]	[5.6.5]	[5.6.5]	[5.6.5]	[5.5.5]	-	[5.6.4]	[5.6.5]
TRBV25	[5.6.4]	[5.6.4]	[5.6.4]	[5.6.4]	[5.6.4]	[5.6.4]	[5.6.4]	[5.6.4]
TRBV26	[5.6.4]	-	[5.6.4]	[5.6.4]	[5.6.4]	[5.6.4]	[5.6.4]	-
TRBV27	[5.6.4]	[5.6.4]	[5.6.4]	[5.6.4]	[5.6.4]	[5.6.4]	-	[5.6.4]
TRBV28	[5.6.4]	[5.6.4]	[5.6.4]	-	[5.6.4]	[5.6.4]	[5.6.4]	[5.6.4]
TRBV29	[5.7.3]	[5.7.3]	[5.7.3]	[5.7.3]	[5.7.3]	[5.7.3]	[5.7.3]	[5.7.3]
TRBV30	[6.5.3]	[6.5.3]	[6.5.3]	[6.5.3]	[6.5.4]	[6.5.3]	[6.5.3]	[6.5.3]

*Only in frame genes are considered. The differences in CDR3-IMGT length are shown in red. The differences in CDR1-IMGT or CDR2-IMGT lengths are shown in green. Data available in IMGT Repertoire (IG and TR) http://imgt.org/IMGTrepertoire/ > 2D and 3D structures > FR-IMGT and CDR-IMGT lengths (V-REGION and V-DOMAIN) > [CDR1-IMGT.CDR2-IMGT.] length per subgroup > TRBV > Human, ibid. Rhesus monkey, ibid. Dog, ibid. Cat, ibid. Ferret, ibid. Rabbit, ibid. Sheep, ibid. Pig*.

On the other hand, the CDR lengths in the other subgroups are relatively well-conserved between the different species (*cf*. Table 3). The most important differences are in germline CDR3-IMGT, indeed the length varies from one or two AA in genomic sequences. These differences are shown in red in Table 3 and correspond to 5 out of 13 TRBV6 genes in rabbit, the TRBV20 gene in ferret, the TRBV21 gene in rhesus monkey, the TRBV22 and the TRBV24 in sheep, and the TRBV30 gene in ferret. There are also insertions and deletions in CDR1-IMGT or CDR2-IMGT as for instance one of the TRBV5 genes, namely in sheep (deletion of CDR1-IMGT), the TRBV6 gene in ferret (insertion of 4 AA in CDR2-IMGT), the TRBV22 in rhesus monkey (deletion of 2 AA in CDR1-IMGT) and the TRBV24 in ferret (deletion of 1 AA in CDR2-IMGT) shown in green in Table 3.

### 3.3. Comparison of the D-J-C-CLUSTER

The number of D-J-C-CLUSTER (which describes set of genes including one TRBD, 6-8 TRBJ and one TRBC gene) differs according to the species. In sheep and pig there is a third D-J-C-CLUSTER between the first and the second D-J-C-CLUSTER (*cf*. Figure 3). There is 1 TRBD, 6 or 7 TRBJ, and 1 TRBC more in these two species which corresponds to the number of genes identified in a D-J-C-CLUSTER (*cf*. Table 1). However, the number of TRBD, TRBJ and TRBC within the three clusters is conserved: 1 TRBD, 6–8 TRBJ and 1 TRBC (*cf*. Table 4). Regarding the functionality, all the TRBD and TRBC genes are functional and few TRBJ genes are pseudogenes (1 gene in dog, in ferret, in sheep and in rhesus monkey, 2 genes in pig, and 3 genes in cat) (*cf*. Table 4).

**Table 4:** IMGT Potential germline repertoires of the TRBJ sets in human (*Homo sapiens*), rhesus monkey (*Macaca mulatta*), dog (*Canis lupus familiaris*), cat (*Felis catus*), ferret (*Mustela putorius furo*), rabbit (*Oryctolagus cuniculus*), sheep (*Ovis aries*), and pig (*Sus scrofa*).

**Sets**	***Homo sapiens***	***Macaca mulatta***	***Canis lupus familiaris***	***Felis catus***	***Mustela putorius furo***	***Oryctolagus cuniculus***	***Ovis aries***	***Sus scrofa***
TRBJ1	6 F (7)	6 F (7)	4 F, 1 O, 1 P	3 F, 1 O, 2 P	3 F, 2 O, 1 P	5 F, 1 O	5 F (6), 1 O	6 F, 1 P
TRBJ2	6 F, 1 O, 1 FO (2)	7 F (8), 1 P (2)	5 F, 1 O	5 F, 1 P	4 F, 2 O	6 F (7)	7 F (8)	6 F
TRBJ3	-	-	-	-	-	-	5 F (6), 1 P (2)	6 F, 1 P
Total per Fct	12 F, 1 O, 1 FO	13 F, 1 P	9 F, 2 O, 1 P	8 F, 1 O, 3 P	7 F, 4 O, 1 P	11 F, 1 O	17 F, 1 O, 1 P	18 F, 2 P
Total genes	14 (16)	14 (17)	12	12	12	12 (13)	19 (23)	20

*For each TRBJ set, in each species, the number of TRBJ genes by functionality and, between parentheses, the number of alleles are shown. F: functional; O: ORF; P: pseudogene; FO: genes with alleles of different functionalities. Data available in IMGT Repertoire (IG and TR) http://imgt.org/IMGTrepertoire/ > Locus and genes > Potential germline repertoires > TRBV, TRBD, TRBJ > Human, ibid. Rhesus monkey, ibid. Dog, ibid. Cat, ibid. Ferret, ibid. Rabbit, ibid. Sheep, ibid. Pig*.

**Figure 6:**
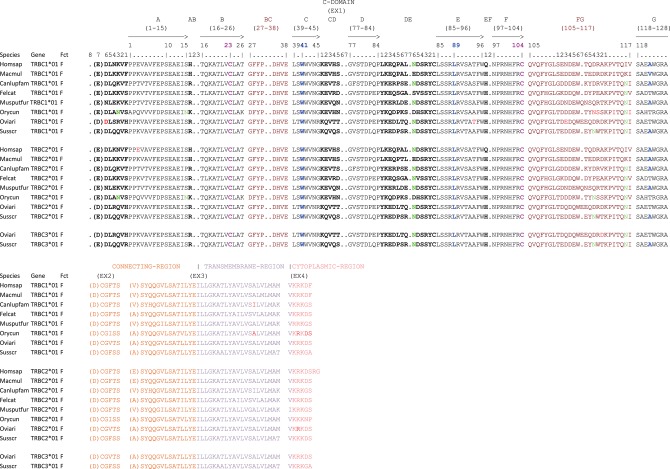
Protein display of the TRBC genes in human (Homsap), rhesus monkey (Macmul), dog (Canlupfam), cat (Felcat), ferret (Musputfur), rabbit (Orycun), sheep (Oviari), and pig (Susscr). The description of the strands and loops is according to the IMGT unique numbering for C-DOMAIN (46) (*cf*. Table S3). The AA between parentheses at the beginning of EX1, EX2 and EX3 corresponds to the first codon resulting from a splicing frame 1 (sf1). The splicing between EX3 and EX4 is a splicing frame 0 (sf0) (http://www.imgt.org/IMGTeducation/Aide-memoire/_UK/splicing/). Data available in IMGT Repertoire (IG and TR) http://imgt.org/IMGTrepertoire/ > Proteins and alleles > Protein displays > C-DOMAIN > TRBC > Human, *ibid*. Rhesus monkey, *ibid*. Dog, *ibid*. Cat, *ibid*. Ferret, *ibid*. Rabbit, *ibid*. Sheep, *ibid*. Pig.

**Figure 7:**
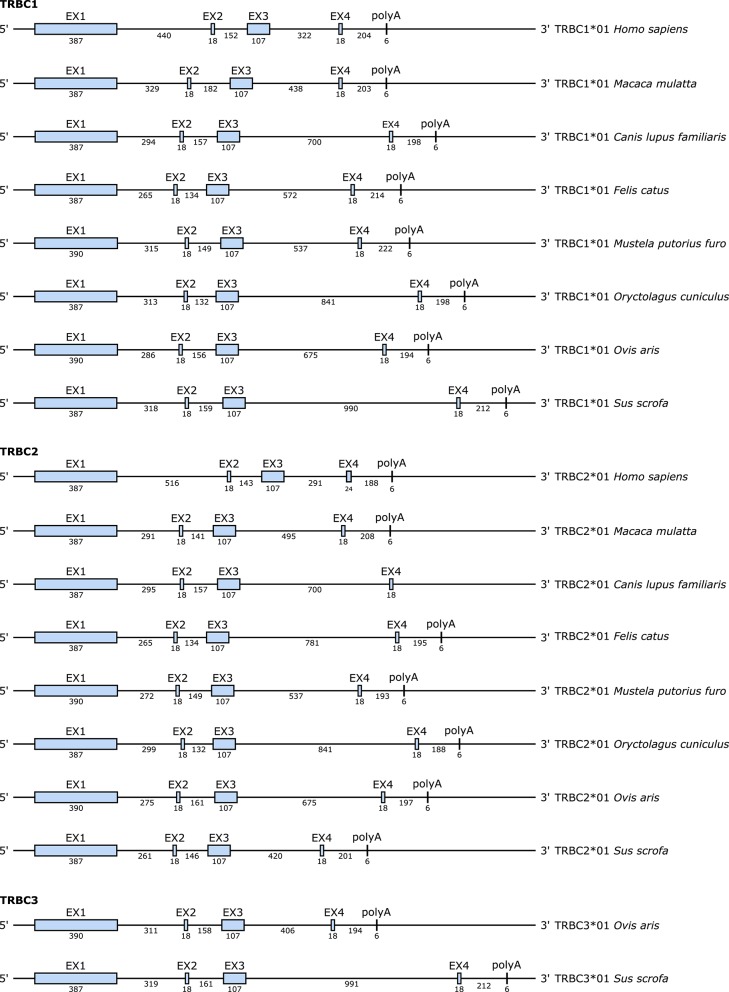
Structure of the TRBC genes in human (*Homo sapiens*), rhesus monkey (*Macaca mulatta*), dog (*Canis lupus familiaris*), cat (*Felis catus*), ferret (*Mustela putorius furo*), rabbit (*Oryctolagus cuniculus*), sheep (*Ovis aries*), and pig (*Sus scrofa*). The numbers correspond to the size of the exons and introns in nucleotides.

At the genomic level, each TRBC gene consists of several exons whose sizes are the same for all species except for exon 1 (EX1) which has an additional AA in the ferret and the sheep at position 112.7 according to IMGT numbering for C-DOMAIN (46) and exon 4 (EX4) in the TRBC2 gene of human (*cf*. Figure 6 and Figure S2). On the other hand, the size of the introns varies according to the species, especially between the exon 3 (EX3) and EX4 (*cf*. Figure 7). Each TRBC gene encodes a similar protein of 176–178 AA, depending on the species, with EX1 encoding the constant domain, the exon 2 (EX2) and the 5′ part of EX3 encoding the connecting region, the 3′ part of EX3 and the first codon of EX4 encoding the transmembrane region and the remaining part of EX4 encoding the cytoplasmic region (*cf*. Figure 6).

## 4. Discussion

This study was carried out in order to compare the TRB locus among seven veterinary species: cat, dog, ferret, pig, rabbit, rhesus monkey and sheep against the human locus. The annotation of each locus followed the pipeline defined in Figure 1. The expertise that follows this pipeline permits to establish the TRB germline repertoire according to IMGT® unique nomenclature and the IMGT® reference directory (IMGT® reference sequences used by IMGT® tools) of each locus and thus obtain sequence, gene and structure data. For each gene analyzed, there are more than 200 pieces of information available in IMGT® databases, tools and web pages. The comparison of the data obtained after the biocuration was carried out against the data of the human TRB locus. This analysis was done with respect to the data entered in IMGT Repertoire.

With the exception of the rabbit locus, the other loci have few, if any, gaps (*cf*. Table S1). Indeed, it is a basic criterion for the annotation of a complete locus with a definitive nomenclature in IMGT. The annotations made correspond either to published publications or to collaborations. We rely on publicly available data, which is why we need good quality data so that we can annotate what we see with good quality annotations.

During the analysis of the TRB locus in different species, it was noted that the general organization of the locus is conserved among the eight species studied. It should be emphasized that the IMGT® unique nomenclature, based on subgroup assignment and position of genes within the locus, represents a quite help for evidence of locus organization similarities. Nevertheless, there are differences depending on the species, especially for the location of the first gene (TRBV1), the number and location of TRY and the number of D-J-C-CLUSTER.

The results show that some subgroups are more represented in rabbit (TRBV5, TRBV6, and TRBV7) or in sheep (TRBV5 and TRBV6) than in other species, which may indicate potential duplications during evolution. It can also explain the difference in the proportion of functional genes. Indeed, duplicated subgroups in rabbit (TRBV5, TRBV6, and TRBV7) are composed mainly of functional genes which makes the functional genes predominant in this species while duplicated subgroups in sheep (TRBV5 and TRBV6) are composed of half of functional genes and half of pseudogenes resulting in similar proportion of pseudogenes and functional genes. One question that might emerge from these results is the following, “what is the diversity of the repertoires of these species according to the F and ORF genes?” Currently, the number of available cDNA sequences in public databases is not large enough to answer this question. The same holds for the detection of genes or subgroups mainly used in rearrangements.

Another indication of duplication during evolution is the third D-J-C-CLUSTER in pig and sheep, also present in bovine (*Bos taurus*), goat (*Capra hircus*), and *Camelus* gender, which highlights a shared evolution in Ruminantia, Suina and Tylopoda (47, 48).

Unlike other loci coding for IG or TR, the CDR lengths do not allow to differentiate the subgroups. Only four subgroups (TRBV1, TRBV20, TRBV29, and TRBV30) have distinct CDR lengths comparing to other subgroups (*cf*. Table 3).

The veterinary species are valuable models for immunological and medical research. The comparison of the TRB locus among several species presented here allow to have a global vision of the TRB locus in vertebrates and will be a useful resource to analyze the TRB locus in new species not yet analyzed. The work carried out and the establishment of the methodology will allow and facilitate the analysis of subsequent TRA, TRD, TRG, IGH, IGK, and IGL loci among different species.

## Data Availability Statement

The datasets generated for this study can be found in the IMGT/LIGM-DB.

## Author Contributions

PP annotated the dog, ferret, rabbit, and sheep TRB locus. MB annotated the rhesus monkey TRB locus. VN annotated the cat TRB locus and IC annotated the pig TRB locus. GF annotated the human TRB locus in 1996 and IC added new alleles according to the last assembly (GRCh38.p12). SH-S and JJ-M, along with all the other biocurators, double checked the final outcomes. VG added the data to IMGT/V-QUEST. PD was in charge of IMGT/HighV-QUEST. M-PL supervised all the annotation projects. PP analyzed the data. PP and SK drafted the manuscript. All the authors read and approved the final manuscript.

## Conflict of Interest

The authors declare that the research was conducted in the absence of any commercial or financial relationships that could be construed as a potential conflict of interest.
