# Supplementary material for: IMGT® Biocuration and Comparative Study of the T Cell Receptor Beta Locus of Veterinary Species Based on Homo sapiens TRB
Source: Front Immunol. 2020 May 5;11:821. doi: 10.3389/fimmu.2020.00821 (PMC7216736; doi:10.3389/fimmu.2020.00821)
Supplement: Supplementary file 4 [file Data_Sheet_1.pdf]

Supplementary Material

1 SUPPLEMENTARY TABLES AND FIGURES

1.1 Figures

Figure S1. Phylogenetic tree of all TRBV genes for all species (using V-REGION). Homsap: human, Macmul: rhesus monkey, Felcat: cat, Canlupfam: dog, Musputfur: ferret, Orycun: rabbit, Susscr: pig and Oviari: sheep. Tree generated using NGPhylogeny.fr (Lemoine et al., 2019) (with MAFFT (Katoh and Standley, 2013) and PhyML (Guindon et al., 2010) programs) and iTOL v4 (Letunic and Bork, 2019).

Figure S2. IMGT Collier de Perles of the TRBC1 gene. A. in human and B. in sheep. The insertion in position 112.7 according to IMGT numbering for C-DOMAIN is framed in red. Amino acids are shown in the one-letter abbreviation. All proline (P) are shown online in yellow. IMGT anchors are in square. Hatched circles are IMGT gaps according to the IMGT unique numbering for C-DOMAIN (Lefranc et al., 2005). Positions with bold (online red) letters indicate the four conserved positions that are common to a V-DOMAIN and to a C-DOMAIN: 23 (1st-CYS), 41 (CONSERVED-TRP), 89 (hydrophobic), 104 (2nd-CYS) (Lefranc et al., 2003, 2005), and position 118, which is only conserved in V-DOMAIN. Data available in IMGT Repertoire (IG and TR) http://imgt.org/IMGTrepertoire/ > 2D and 3D structures > Colliers de Perles > C-DOMAIN > TRBC > Human, *ibid*. Sheep

1.2 Tables

Table S1. Information regarding the genome assembly and TRB locus IMGT 5' and 3' borne in human (*Homo sapiens*), rhesus monkey (*Macaca mulatta*), dog (*Canis lupus familiaris*), cat (*Felis catus*), ferret (*Mustela putorius furo*), rabbit (*Oryctolagus cuniculus*), sheep (*Ovis aries*) and pig (*Sus scrofa*).

Table S2. Differences between the data indicated in the articles and the annotated data (number of genes and functionality) in rhesus monkey (*Macaca mulatta*), dog (*Canis lupus familiaris*), cat (*Felis catus*), ferret (*Mustela putorius furo*), rabbit (*Oryctolagus cuniculus*), sheep (*Ovis aries*) and pig (*Sus scrofa*).

Table S3. Strand and loop length of the TRBC genes inhuman (*Homo sapiens*), rhesus monkey (*Macaca mulatta*), dog (*Canis lupus familiaris*), cat (*Felis catus*), ferret (*Mustela putorius furo*), rabbit (*Oryctolagus cuniculus*), sheep (*Ovis aries*) and pig (*Sus scrofa*).

REFERENCES

Guindon, S., Dufayard, J.-F., Lefort, V., Anisimova, M., Hordijk, W., and Gascuel, O. (2010). New algorithms and methods to estimate maximum-likelihood phylogenies: assessing the performance of PhyML 3.0. Systematic Biology 59, 307–321. doi:10.1093/sysbio/syq010

Katoh, K. and Standley, D. M. (2013). MAFFT multiple sequence alignment software version 7: improvements in performance and usability. Molecular Biology and Evolution 30, 772–780. doi:10.1093/molbev/mst010

Lefranc, M.-P., Pommié, C., Kaas, Q., Duprat, E., Bosc, N., Guiraudou, D., et al. (2005). IMGT unique numbering for immunoglobulin and T cell receptor constant domains and Ig superfamily C-like domains. Developmental and Comparative Immunology 29, 185–203. doi:10.1016/j.dci.2004.07.003

Lefranc, M.-P., Pommié, C., Ruiz, M., Giudicelli, V., Foulquier, E., Truong, L., et al. (2003). IMGT unique numbering for immunoglobulin and T cell receptor variable domains and Ig superfamily V-like domains. Developmental and Comparative Immunology 27, 55–77

Lemoine, F., Correia, D., Lefort, V., Doppelt-Azeroual, O., Mareuil, F., Cohen-Boulakia, S., et al. (2019). NGPhylogeny.fr: new generation phylogenetic services for non-specialists. Nucleic Acids Research 47, W260–W265. doi:10.1093/nar/gkz303

Letunic, I. and Bork, P. (2019). Interactive Tree Of Life (iTOL) v4: recent updates and new developments. Nucleic Acids Research 47, W256–W259. doi:10.1093/nar/gkz239
