# Supplementary figures and images for: IMGT® Biocuration and Comparative Study of the T Cell Receptor Beta Locus of Veterinary Species Based on Homo sapiens TRB
Source: Front Immunol. 2020 May 5;11:821. doi: 10.3389/fimmu.2020.00821 (PMC7216736; doi:10.3389/fimmu.2020.00821)

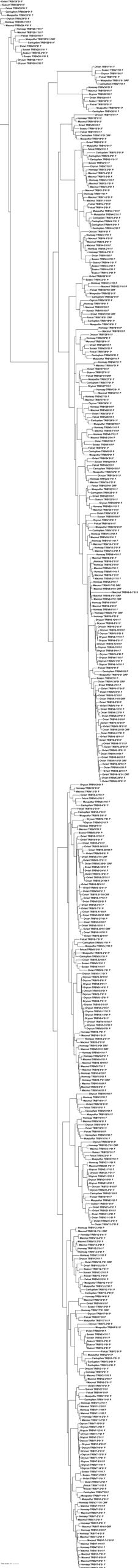

Supplement: Supplementary file 5 [file Image_1.PDF]

## On one layer

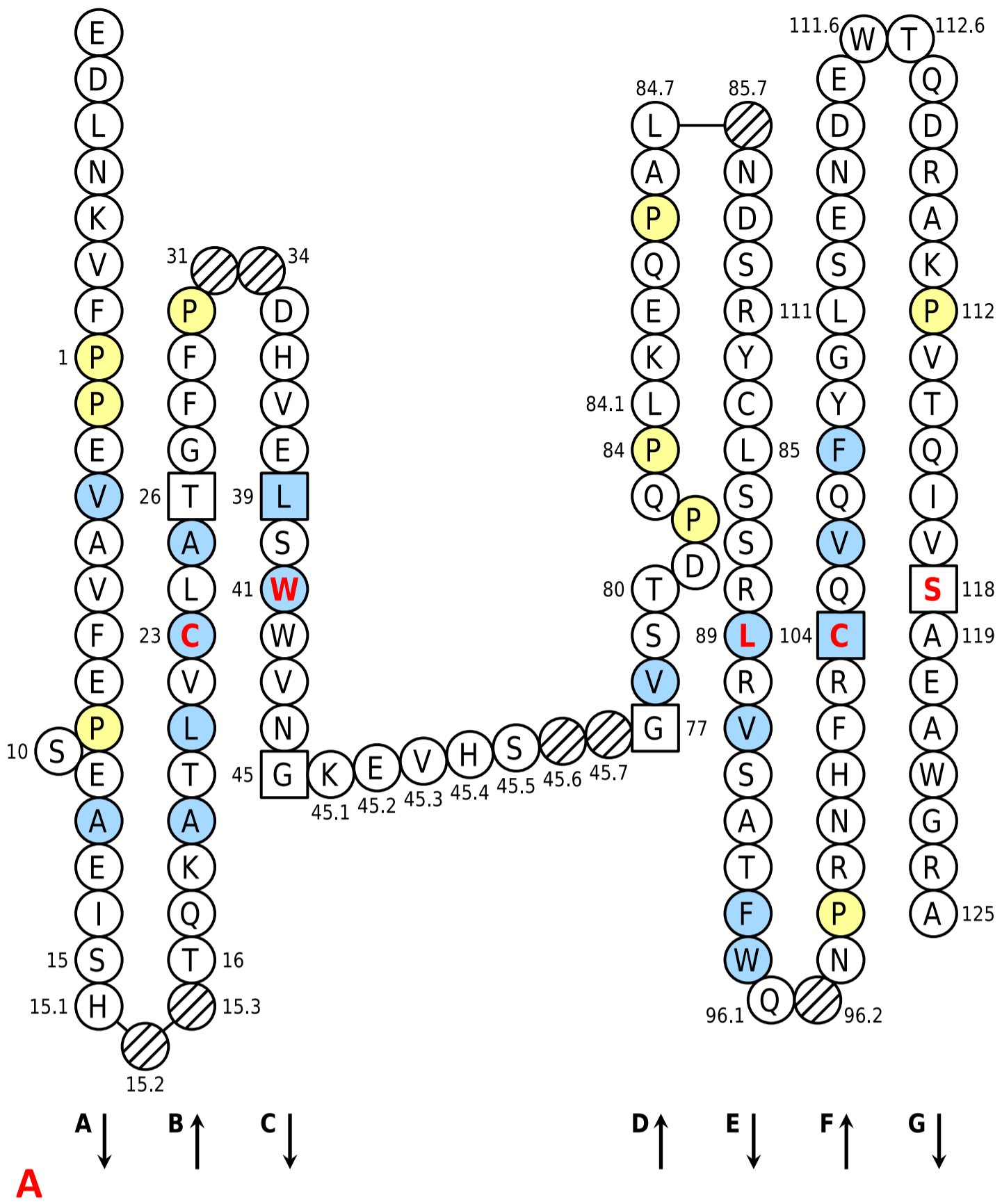

## On two layers

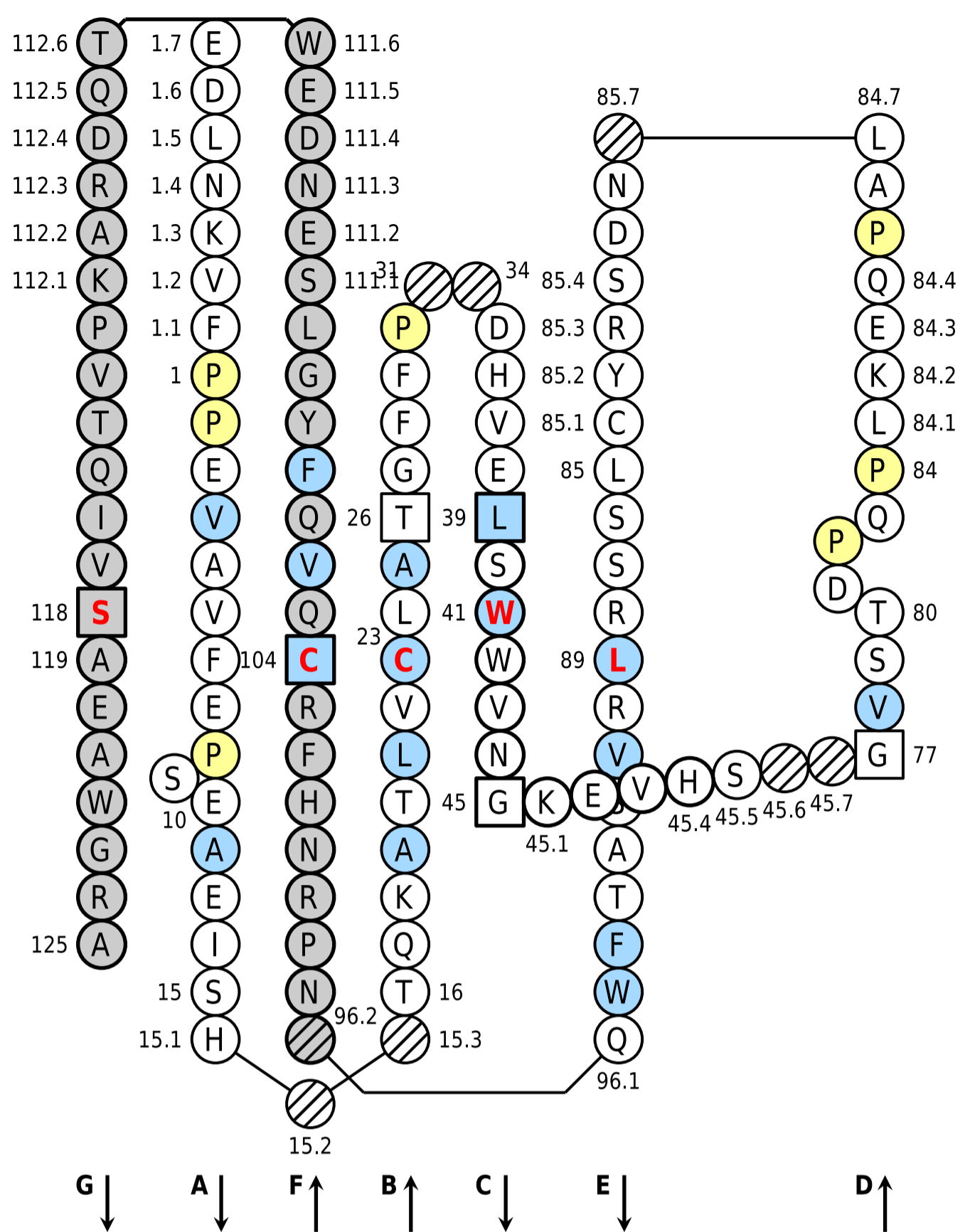

## On one layer

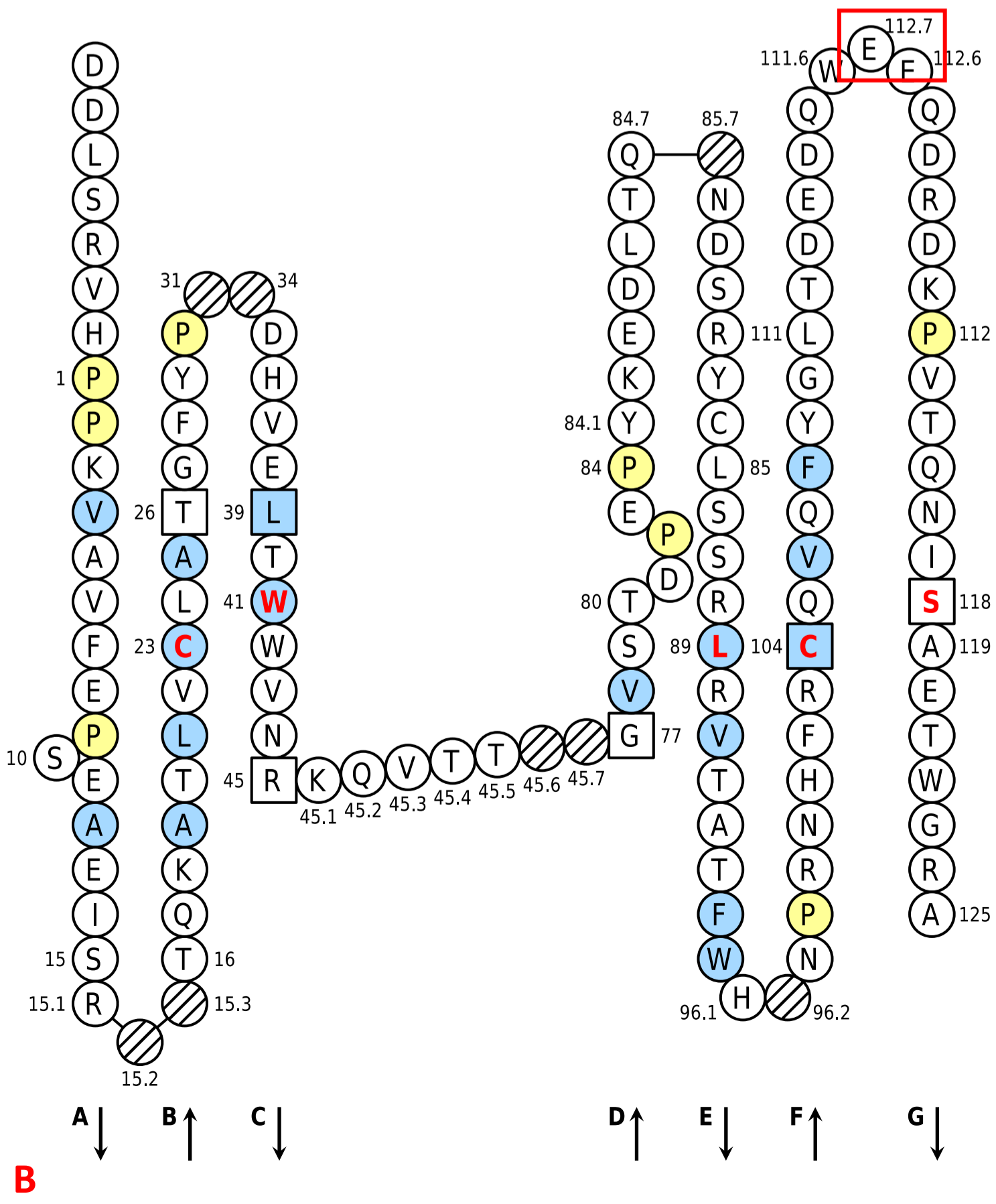

## On two layers

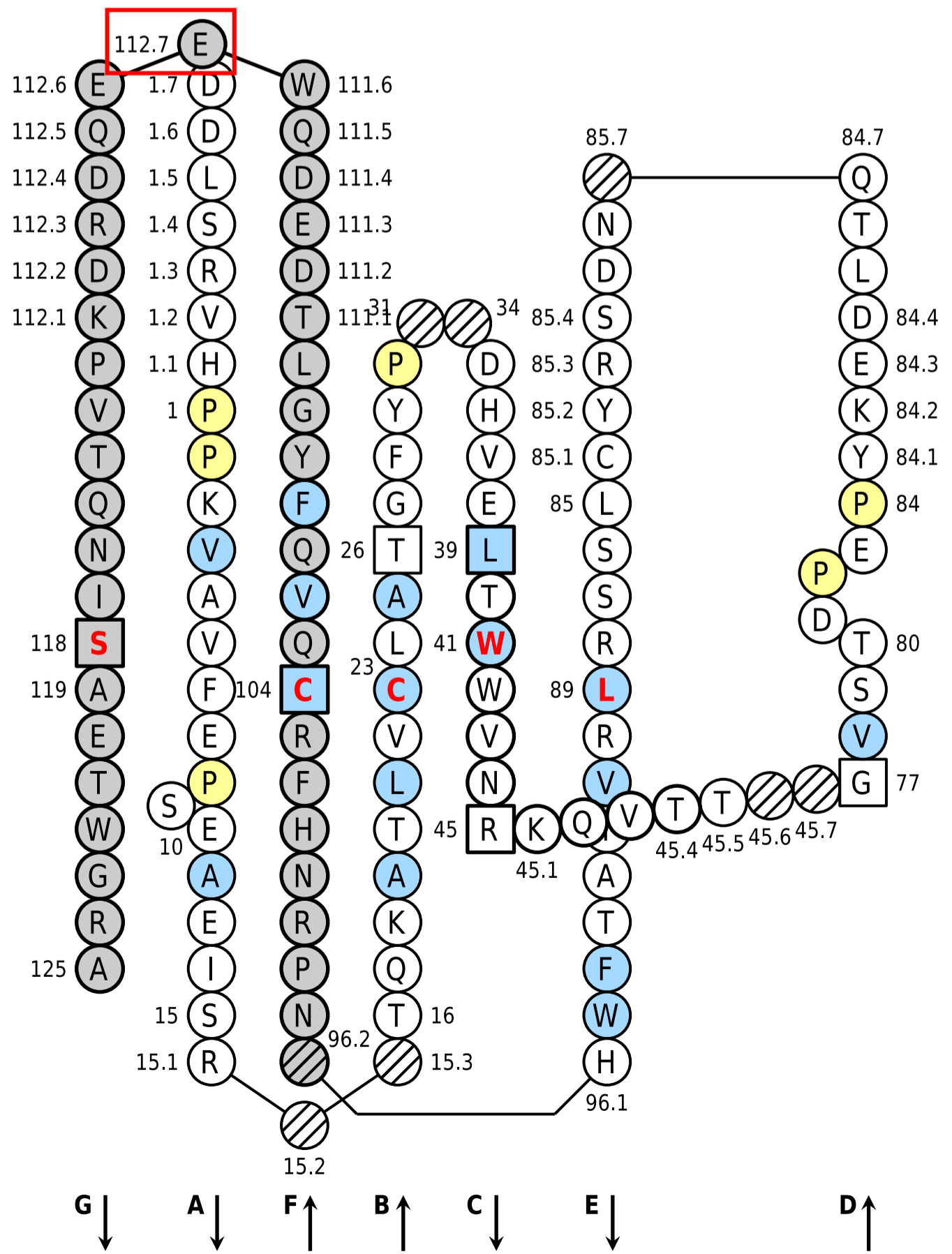

Supplement: Supplementary file 6 [file Image_2.PDF]
